# Functional analysis of 4-coumarate: CoA ligase from *Dryopteris fragrans* in transgenic tobacco enhances lignin and flavonoids

**DOI:** 10.1590/1678-4685-GMB-2018-0355

**Published:** 2020-05-20

**Authors:** Shan-Shan Li, Ying Chang, Bo Li, Shu-Li Shao, Zhen-Zhu-Zhang

**Affiliations:** 1 Qiqihar University, College of Life Sciences and Agriculture and Forestry, Qiqihar, China,; 2 Heilongjiang Provincial Key Laboratory of Resistance Gene Engineering and Protection of Biodiversity in Cold Areas, Qiqihar, China.; 3 Northeast Agricultural University Laboratory of Plant Research College of Life sciences, Harbin, China.

**Keywords:** phenylpropanoid metabolic pathway, 4-coumaric acid: coenzyme A ligase, ferns, Dryopteris fragrans, metabolites

## Abstract

4-Coumaric acid: coenzyme A ligase (4CL) is a key enzyme in the phenylpropanoid metabolic pathway that regulates the biosynthesis of lignin and flavonoids. Therefore, the study of 4CL is important to explore the accumulation and regulation of metabolites. This study investigated the role that the *4CL2* gene from *Dryopteris fragrans* (*Df4CL2*) plays in the metabolite synthesis. Changes in gene expression, enzyme activity, and the content of lignin and flavonoids were measured in different tissues of tobacco as model plant that was successfully transferred with *Df4CL2*. Tobacco plants with *Df4CL2* (transgenic tobacco, TT) were successfully obtained via the Agrobacterium-transformation method. This TT tended to be thicker and had an earlier flowering period than wild type tobacco (WT). The expression levels of *Df4CL2* were higher in the stem, leaf, and root in TT compared to WT. In addition, compared to WT, TT had higher 4CL enzyme activity and higher lignin and flavonoids contents. This suggests that *Df4CL2* is involved in the synthesis of lignin and flavonoids in D. fragrans. This research provides important evidence toward understanding the phenylpropanoid metabolic pathway in ferns.

## Introduction

The secondary metabolic pathways of phenylpropanoid have been studied in detail, especially in angiosperms, where the metabolic pathway has been explored in detail (Dixon *et al.*, 2002; Hamberger and Hahlbrock, 2004). In this particular metabolic pathway, 4-Coumaric acid: coenzyme A ligase (4CL; EC 6.2.1.12) is a key enzyme, located in the transition from the general pathway to the downstream branch pathway (Vassão *et al.*, 2010; Gui *et al.*, 2011). 4CL uses cinnamic acid, 4-coumaric acid, 5-hydroxyferic acid, sinapic acid, caffeic acid, and ferulic acid as substrates to form the corresponding acyl-CoA thioester in the presence of ATP and Mg^2+^. These become substrates in different downstream branch pathways. 4CL generally exists as an isozyme in different plants and has different substrate preferences. It regulates the amount of CoA esters by catalyzing different substrates, or it enters the lignin biosynthesis-specific pathway through a reaction catalyzed by cinnamoyl-CoA reductase (CCR) and cinnamyl alcohol dehydrogenase (CAD). 4CL can also enter flavonoids through either chalcone synthase (CHS) or chalcone isomerase (CHI) (Harding *et al.*, 2002). Recent studies indicated that 4CL is one of the rate-limiting enzymes in the phytochemical monomer synthesis pathway of vascular plants. This has also become a focus in genetic engineering research to regulate the lignin content of vascular plants (Emiliani *et al.*, 2009; Rao *et al.*, 2015; Lavhale *et al.*, 2018; Liu *et al.*, 2018). Therefore, the in-depth study of the *4CL* gene and enzyme activity not only has an important role in the process of plant growth and development, as well as in growth in adverse environments, but it also provides an important guidance in the production and regulation of secondary metabolites (Blach-Olszewska *et al.*, 2008). 4CL has been studied in angiosperms, gymnosperms, and bryophytes; however, in ferns, its metabolic pathway and function remains not clearly identified.

*Dryopteris fragrans* is a perennial medicinal fern that is widely distributed in the Heilongjiang Province of China, especially in the magma gap around the volcanos in the Wudalianchi Scenic Area. The unique characteristics of this growth environment confer unique medicinal value on *D. fragrans*. As a folk medicine, *D. fragrans* is mainly used to treat arthritis and various skin diseases, including psoriasis, acne, rash, and dermatitis. Chinese and foreign researchers found that *D. fragrans* also has multiple features including deworming, antibacterial (Shen *et al.*, 2005; Zhang *et al.*, 2008a), antioxidant (Zhang *et al.*, 2008b; Li *et al.*, 2012a), analgesic (Wang *et al.*, 2008), and antitumor (Ito *et al.*, 2000; Li *et al.*, 2012b) activities. A major class of functional substances contained in *D. fragrans* are flavonoids. The metabolic pathways and key enzymes resulting from the synthesis of compounds have become a major breakthrough in regulating these effective substances. The synthetic pathway of metabolites in ferns has not yet been fully understood, and the function of key enzymes remains unknown. Therefore, it is important to explore the synthesis and regulation of both flavonoids and lignin in metabolites from the genetic perspective.

In this study, the *Df4CL2* gene of *D. fragrans* was transferred into the tobacco model plant, and the relationship between 4CL2, flavonoids, and lignin was identified by examining the genetic expression, enzyme activity, and metabolites. In doing so, a basis was provided for the identification of the phenylpropanoid metabolic pathway of this fern and for the extensive application of this species.

## Material and Methods

### Genetic transformation of the gene

The *Df4CL2* (KF836752) gene obtained from prior experiments was used to construct the pBI121*-Df4CL2* expression vector (Li *et al.*, 2015). The genetic transformation of tobacco was performed through the Agrobacterium-mediated transformation as described by Hoekema *et al.* (1983). T_1_-generation-regenerated kanamycin-resistant plants were identified using PCR, and successfully identified plants were reserved for subsequent experiments. The specific primers for PCR identification were: 4CL-F, 5’-GGCTGAGGTCCTTCCCTCCTCTG-3’, 4CL-R,5’-ACTAGCGCTATTTGATTTTCTTAATGC-3’. PCR parameters were: initial denaturation for 5 min at 95 °C, followed by 35 cycles of denaturation for 30 s at 95 °C, annealing for 30 s at 58 °C, and extension at 72 °C for 2 min, with a final extension step for 10 min at 72 °C. The PCR products were observed via 1% agarose gel electrophoresis (Li *et al.*, 2015).

### Expression of *Df4CL2* in transgenic tobacco

RNA from root, stem, leaf, flower, and seed of TT was extracted using the RNA plant Plus Reagent (TIANGEN, Beijing, China) according to the manufacturer’s protocol. Reverse transcription was performed using the Transcriptor First Strand cDNA Synthesis Kit (HaiGene, Harbin, China). Real-time PCR was performed on an ABI Prism 7500 sequence detector using the manufacturer’s THUNDERBIRD SYBR qPCR Mix (Toyobo, Osaka, Japan). The utilized primers were: 18srRNA-F, 5’-TTGACGGAAGGGCACCA-3’,18srRNA-R5’-ACCACC ACCCATAGAATCAAGAA-3’,4CL2-qPCR-F5’-CTCATCGAGCACAGCAACACTGAATTCAA-3’,4CL2-qPC R-R 5’-CTGGTACAACAGGCTTACCCAAAGGAGTC-3’. The PCR assay was performed as follows: 95 °C for 1 min, and 40 cycles of 95 °C for 15), 60 °C for 35 s, and 72 °C for 20 s (Li *et al.*, 2015). The relative expression was analyzed according to the 2^-ΔΔCT^ method (Livak and Schmittgen, 2001).

### Assay of 4CL enzyme activity

The protein content of extracts from the root, stem, leaf, and flower of TT was assayed using the Coomassie brilliant blue method. The enzyme activity was assayed according to the instructions of the 4-coumaric acid: coenzyme A ligase (4CL) enzyme-linked immunoassay kit (Beijing GDELISA Biotechnology Co., Ltd., Beijing, China). The specific activities of 4CL were calculated as: Enzyme activity (IU) / Protein (mg).

### Assay of lignin content

A qualitative study was performed using phloroglucinol-HCl staining. A cross section of the main tobacco stem was placed on a glass slide for immediate phloroglucinol-HCl staining. The sections were observed under a microscope, and then photographed.

Fresh seedlings grown for 10 days were taken and the lignin content was measured according to the method by Fukushima and Hatfield (2001, 2004). The absorbance value was measured at 280 nm, with A280 nm mg protein^-1^ representing the lignin content.

Roots, stems, leaves, flowers, and seeds of tobacco were taken, and the lignin content of mature tobacco was determined according to the method described by Romualdo *et al.* (2001), with the absorbance value being measured at 280 nm.

$$Lignin\%=\frac{Abs\times liters\times 100\%}{W_{sample}\times A_{standard}}$$

Lignin%: lignin content; Abs: absorbance value of the sample solution at 280 nm, Liters: volume (L) of the sample solution, W_sample_: absolute dry weight of the sample (g), A_standard_: standard absorbance of lignin in tobacco, A_standard_ = 20.0.

### Assay of flavonoids

Flavonoids were extracted according to the method of Burbulis *et al.* (1996), and the extracted samples were stored at 4 °C, or retained for HPLC analysis. HPLC analysis was performed on a chromatography workstation, comprising a universal sampler (model U6K; Waters), a dual pump system (model 510; Waters, Bedford, MA), an automatic gradient controller (model 680; Waters), a photodiode array detector (model 484; Waters), and a data module (model 745B; Waters). Samples were loaded on C18 reversed-phase columns (Nova-Pak 60A, 4 μm, 3.9 x 75 mm) at 25 °C at a flow rate of 2.5 mL/min. The eluate was collected and detected at 255 nm. Kaempferol (Sigma) dissolved at 0.1 mg/mL in 80% methanol was used as standard sample. The retention time of the samples was compared to the standard sample. The monomer concentration in the test agent was assayed via the integral of the area of the eluting peak.

### Statistical analysis

All experiments were performed in at least three biological replicates, and the data were analyzed using SPSS 20.0 software.

## Results

### Identification of T_1_-generation transgenic tobacco

The expression vector pBI121-*Df4CL2* was transfected into a sterile tobacco leaf by the Agrobacterium *EHA105* to induce s callus, and the T_0_-generation of TT plants was then cultivated. T_0_-generation kanamycin-resistant plants were screened by PCR, and seeds were obtained from successfully transferred plants. The T_1_-generation of TT obtained from seeds was also screened by PCR, and the successfully transferred plants were reserved for subsequent experiments.

**Figure 1:**
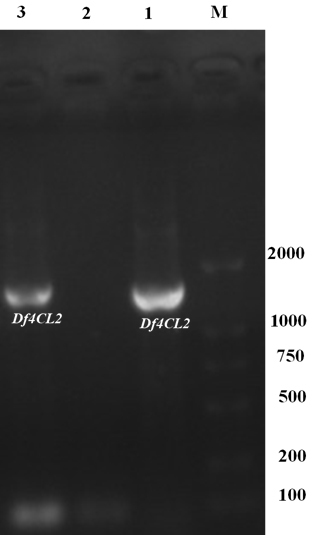
PCR amplification of *Df4CL2* in T_1_-generation. Lanes: 1 and 3, TT; 2, WT; M, marker.

As shown in Figure 1, a amplicon band was found at 1700 bp, which was consistent with the predicted length of *Df4CL2*. This indicates that the *Df4CL2* gene was successfully transferred into tobacco.

### State of growth in transgenic tobacco

**Figure 2:**
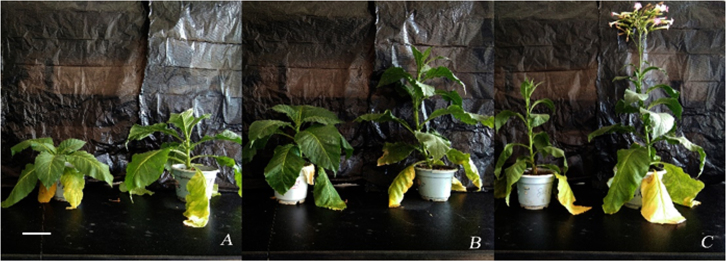
Growth and development of tobacco in different periods. TT and WT grown for 90 (A), 120 (B), and 150 days (C). The left plant is WT, the right plant is TT. Bar size is 11.5 cm.

The morphological characteristics and growth of the T_1_-generation of both TT and WT were observed at 90 d, 120 d, and 150 d, respectively. As shown in Figure 2, TT was 1.7-, 1.8-, and 1.5-fold higher than WT at 90 d (Figure 2A), 120 d (Figure 2B), and 150 d (Figure 2C), respectively. In addition, the number of leaves was increased by 1.3-, 1.7-, and 1.3-fold, respectively. Compared to WT, the flowering period of TT was initiated 14 days earlier.

### Expression of *Df4CL2* in different tissues of transgenic tobacco

The expression levels of *Df4CL2* from different tissues (root, stem, leaf, flower, and seed) in TT were analyzed using fluorescent quantitative PCR with 18S rRNA as internal control.

**Figure 3:**
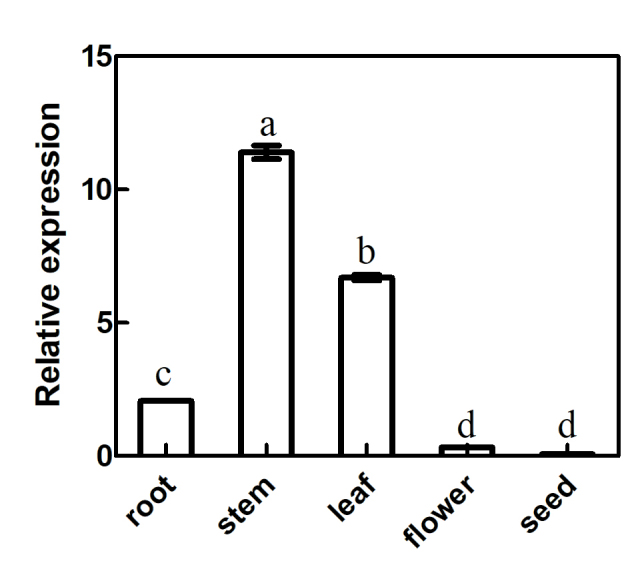
Analysis of *Df4CL2* expression levels in TT using Real-time PCR and 18S rRNA as internal control. Each histogram represents the mean of three biological replicates. Means and standard deviations (SD) (n = 3) of at least three independent experiments are reported. Different letters indicate significant difference (*p* < 0.05, one-way ANOVA, followed by Duncan’s test).

As shown in Figure 3, the expression levels of Df4CL2 in TT can be arranged in a descending order: stem > leaf > root > flower > seed. Its expression level in the stem (which was the tissue with the highest expression) was 1.7-fold higher than in the leaf, 5.5-fold higher than in the root, 34-fold higher than in the flower, and 164-fold higher than in the seed.

## Analysis of 4CL enzyme activity

**Figure 4:**
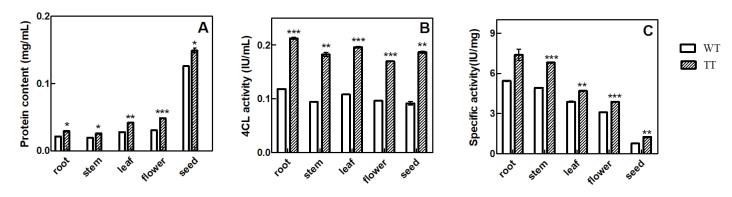
Analysis of enzyme activity of 4CL in the different tissues of WT and TT. (A) Protein content; (B) enzyme activity; (C) specific activity. The values are presented as means ± standard deviations (SD) (n = 3). *, **, *** indicate significant differences in comparison to the WT plants at *p* < 0.05, v < 0.01, and *p* < 0.001, respectively (Student’s *t*-test).

The protein contents in different tissues of TT and WT were assayed by the Coomassie Brilliant Blue method. As shown in Figure 4A, compared to WT, in TT the protein content was found increased at different levels. The protein concentration (mg/mL) in the root, stem, leaf, flower, and seed was increased by 1.36-,1.33-, 1.48-, 1.56-, and 1.81- fold, respectively.

The 4CL enzyme activities in the root, stem, leaf, flower, and seed from WT and TT were measured with the plant 4-coumaric acid: coenzyme A ligase enzyme-linked immunoassay kit. As shown in Figure 4B, the enzyme activity of 4CL in TT was enhanced in all tissues. Compared to WT, in TT, the enzyme activity increased by 1.8-, 1.93-, 1.81-, 1.96-, and 1.97-fold in the root, stem, leaf, flower, and seed, respectively. This increase in enzyme activity should enhance the reaction rate of the metabolic pathway and promote the synthesis of metabolites.

The specific activities in different tissues in TT and WT were also analyzed. As shown in Figure 4C, the 4CL specific activities in all TT tissues were higher than those of WT. The increase in the root, stem, leaf, flower, and seed was 1.34-, 1.40-, 1.21-, 1.25-, and 1.67-fold, respectively. A higher specific activity indicates a higher enzyme activity.

## Analysis of lignin content

**Figure 5:**
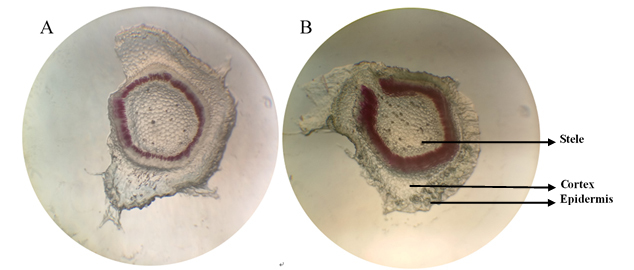
Cross section of main stems from WT (A) and TT (B) seedlings, stained with phloroglucinol-HCl. The red part is the xylem of the main stem.

To test the function of *Df4CL2*, the lignin content of seedlings and mature plants was analyzed and assayed by phloroglucinol staining of cross sections of the tobacco stem. As shown in Figure 5, the outer layer showed the epidermis and the cortex of the main stem. In the central part of the stele, the red part stained by phloroglucinol is the xylem. This shows that, compared to WT, the color of the TT xylem was darker, indicating that the lignin content of TT was higher.

**Figure 6:**
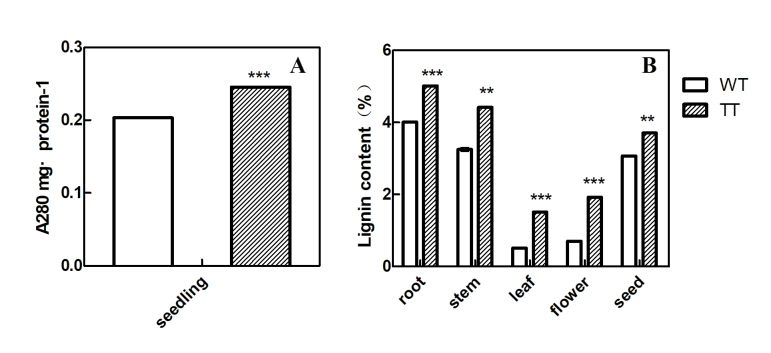
Lignin content in WT and TT plants. Whole seedlings were obtained at 15 days (A), and different tissues were obtained at 120 days (B), respectively. The values and error bars indicate the mean and standard deviations (SD) (n = 3), respectively, from three independent measurements. ** and *** indicate significant differences in comparison to the WT plants at *p* < 0.01 and *p* < 0.001, respectively (Student’s *t-*test).

In addition, the lignin contents in seedling and the root, stem, leaf, flower, and seed were assayed for mature WT and TT plants. As shown in Figure 6, compared to WT, the lignin content in TT seedling was increased by 1.22- fold. In mature plants, the root, stem, leaf, flower, and seed tissues showed an increase in lignin content by 1.25-, 1.37-, 2.0-, 1.70-, and 1.19-fold, respectively.

## Analysis of flavonoid content in different tissues of transgenic tobacco

**Figure 7:**
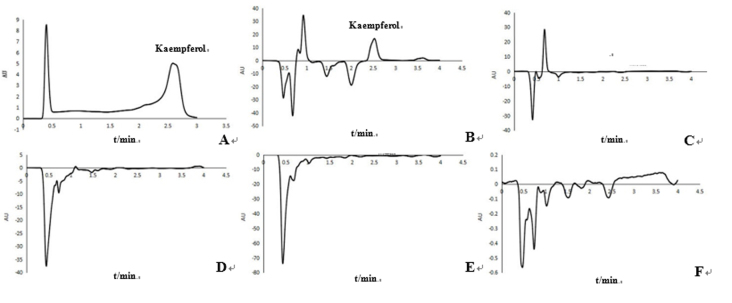
Kaempferol content in TT and WT determined by HPLC. (A) Kaempferol standard; (B) flower; (C) root; (D) stem; (E) leaf; (F) seed. The peak time of kaempferol standard was 2.5 min.

**Figure 8:**
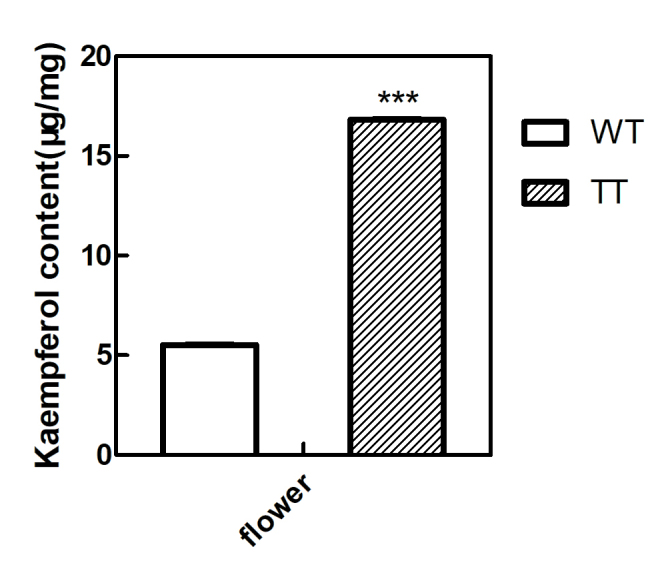
Content of kaempferol in WT and TT plant flowers determined by HPLC. The values and error bars indicate the mean and standard deviations (SD) (n = 3), respectively, from three independent measurements. *** indicates significant differences in comparison to the WT plants at *p* < 0.001, respectively (Student’s *t*-test).

The kaempferol contents in the root, stem, leaf, flower, and seed of TT were assayed by HPLC. As shown in Figure 7, the peak time of kaempferol in the TT flower was identical to the standard sample at 2.5 min; however, there was no peak for the root, stem, leaf, and seed at this time. Kaempferol detected in the TT flower tissue was 3-fold higher than in the WT flower (Figure 8).

## Discussion

About one fifth of the secondary metabolites in plants are produced by the phenylpropanoid metabolic pathway (Dixon *et al.*, 2002; Hamberger and Hahlbrock, 2004). These secondary metabolites not only have multiple physiological functions in plants, but also play an important role in maintaining human health (Jorgensen *et al.*, 2005). For example, the flavonoid rutin produced by this metabolic pathway can effectively reduce the fragility and improve the permeability of blood vessels. Therefore, this metabolic pathway has become a research focus, particularly for the production and regulation of secondary metabolites and on the regulatory mechanism of key genes.

The phenylpropanoid metabolic pathway in angiosperms has been reported in detail (Lavhale *et al.*, 2018); however, its function in ferns remains unclear. Whether the synthesis and accumulation of these metabolites in ferns have the same or similar metabolic pathways as those of angiosperms remains to be explored. Therefore, this study investigated the key enzyme 4CL in the phenylpropanoid metabolic pathway in angiosperms and explored the relationship between Df4CL2 and metabolites in the fern *D. fragrans*.

In *Df4CL2* transgenic tobacco (TT), the expression levels of *Df4CL2* in the stem, leaf, and root were higher than in wild type (WT). In these tissues, compared to WT, the enzyme activity, as well as the lignin content was increased. This indicates that *Df4CL2* was involved in lignin synthesis. In addition, although the expression level of *Df4CL2* was not high in the seed and leaf, it was found to be expressed in these tissues of TT, and compared to WT, its protein content, enzyme activity, specific activity, as well as the lignin content were increased. In addition, in the flower, the content of flavonoids was significantly increased compared to kaempferol used as standard sample.

From this, it can be concluded that *Df4CL2* is involved in the synthesis of lignin and flavonoids. However, several problems remain. For example, the expression levels of *Df4CL2* in different tissues are not consistent with the expression trends of the respective protein, enzyme activity, specific activity, and lignin content. Therefore, a literature search was conducted. First, since the genes in ferns were transferred to tobacco plants, these can express enzymatic activity. While, the enzyme activity of 4CL is substrate-specific, the enzyme activity of Df4CL2 in tobacco plants may be affected by the contents of different substrates in different tissues. Different substrate affinities may direct metabolic flux via different pathways for the synthesis of a variety of phenolic compounds, including different monolignols, flavonoids, isoflavonoids, coumarins, and suberin (Naoumkina *et al.*, 2010). Secondly, in specific plants, 4CL is functionally differentiated, e.g., in Arabidopsis, At4CL3 is responsible for the synthesis of flavonoids, and At4CL1 and At4CL2 are responsible for the synthesis of lignin (Costa *et al.*, 2003, 2005). However, 4CL in specific plants showed no differentiation of gene functions, which are responsible for the synthesis of lignin and flavonoids (member of 4CL gene family of *P. patens*). Based on the current data analysis, and compared to WT, overexpressed tobacco showed an increase of lignin and flavonoid contents, which indicates that the function of the gene *Df4CL2* in *D. fragrans* is related to the synthesis of lignin and flavonoids. Therefore, gene expression is not necessarily related to the specific metabolite content.

For *O. basilicum*, Rastogi *et al.* (2013) reported that RNAi suppression of Os4CL in leaves leads to a reduction in leaf eugenol content and trichome transcript levels. Considerable increases in endogenous 4-coumarate, ferulate, cinnamate, and caffeate were found, while the lignin content remained unaffected.

In the present study, compared to WT, the content of lignin and flavonoids in TT was increased from the measured substance content. With regard to the growth state of the plants, TT was higher than the WT plants, the stalk was thicker, the leaves were more numerous, and the biomass of TT was higher, which should help the plants to resist adverse conditions by enhancing its lodging resistance (Moura *et al.*, 2010). The increase in lignin content is also important for bio-fibers, as well as for biofuel products (Kim *et al.*, 2019). Kaempferol was used as an example to determine the increase in the content of flavonoids, which is crucial for ferns, and they are important substances that play a role in folk medicine.

In summary, our experiments showed that Df4CL2 is involved in the biosynthesis of lignin and flavonoids, and might not have a differentiated gene function. The biosynthesis and accumulation of flavonoids and the lignin content in *D. fragrans* can be promoted by enhancing the genetic expression of Df4CL2, which lays a foundation for the extensive application of *D. fragrans* in medicinal use.
